# Knowing Is Not Eating: Child-Reported Food Perception Versus Actual Dietary Intake in Southern Peru

**DOI:** 10.3390/nu18193204

**Published:** 2026-09-28

**Authors:** Karina Eduardo, Erick Saldaña, Leon Cutipa

**Affiliations:** 1 Facultad de Negocios, Universidad Tecnológica del Perú, Av. Tacna y Arica 160, Arequipa 04001, Peru; lcutipa@utp.edu.pe; 2 Sensory Analysis and Consumers Study Group, Escuela Profesional de Ingeniería Agroindustrial, Universidad Nacional de Moquegua, Prolongación Calle Ancash s/n, Moquegua 18001, Peru; esaldanav@unam.edu.pe

**Keywords:** food perception, free-listing, 24-h recall, cultural salience, children’s eating behavior, Peru

## Abstract

**Background/Objectives:** Understanding whether children’s perceptions of food, that is, what a child recognizes as healthy, are associated with their actual consumption is key to designing effective nutritional interventions, but this has rarely been directly tested in the children themselves. This study aimed to determine whether children’s food perceptions are associated with their actual recent intake. **Methods:** As secondary objectives, the cultural salience of foods perceived as healthy and unhealthy was characterized in 129 schoolchildren (52 girls, 77 boys; ages 8–12) at a school in Moquegua, southern Peru, using an open-ended listing task (five healthy foods and five unhealthy foods) and Smith’s salience index, and this perception was compared with their reported intake from a 24-h recall (breakfast and lunch; *n* = 128), classified according to NOVA criteria and the Peruvian Dietary Guidelines. **Results:** Apples (S = 0.334) and soda (S = 0.312) were the foods with the highest salience in the healthy and unhealthy domains, respectively. Notably, 85.2% of the children reported at least one healthy food in their actual intake, and 38.3% reported at least one unhealthy food. No association was found between food perception and actual consumption (U = 1072.0, *p* = 0.633); this result was unaffected by the order in which the instruments were administered (χ^2^ = 0.000, *p* = 1.000) and showed no significant differences by sex (*p* > 0.05 in all comparisons). **Conclusions:** These findings did not provide evidence of an association between children’s nutritional knowledge and their actual eating habits in this sample and suggest that nutrition education programs targeting schoolchildren in the Peruvian Southern Andes should not assume, based solely on these findings, that teaching children to recognize healthy foods is necessarily sufficient to change their actual diet.

## 1. Introduction

Accurately assessing children’s dietary intake remains one of the greatest methodological challenges in pediatric nutrition. Tools such as the 24-h recall allow for the reconstruction of a child’s recent food consumption, but their validity depends largely on whether the information comes from the child themselves or from an adult reporting on their behalf, since the two sources do not always agree [1]. At the same time, understanding what a child perceives as “healthy” (a cognitive dimension distinct from what the child actually consumes) is key to designing nutritional interventions that align with how children actually construct their food categories, a process that begins at an early age and is mediated by exposure and the family environment rather than by formal nutritional knowledge [2,3,4]. Furthermore, evidence on school nutrition programs suggests that components that directly modify the food environment (for example, the availability of fruits and vegetables) tend to have more consistent effects on actual consumption than purely educational components focused on nutritional knowledge [5].

This distinction between perception and actual consumption is particularly relevant in populations where child malnutrition and childhood overweight coexist—a pattern that is increasingly documented in low- and middle-income countries as the availability of ultra-processed foods expands, even in contexts with limited access to a varied diet [6]. Specifically, in the case of Peru, a population-based analysis of more than 129,000 mother-child pairs showed that, although child malnutrition was cut in half between 1996 and 2016, the likelihood that a child lives with an overweight or obese mother has increased steadily over the same period, regardless of the child’s nutritional status [7]. Peru is no exception to this trend; one study showed that children who consume sugary drinks and snacks daily experience significantly greater weight gain over time [8], while household-level consumption analyses indicate that the Peruvian diet continues to rely heavily on ultra-processed and refined foods [9].

The southern Peruvian Andes, in particular, combine altitudinal, cultural, and socioeconomic conditions that distinctly shape food availability and family dietary practices. Our research group has previously explored this region from an intergenerational perspective: a study combined a structured food classification task with children from Moquegua and Puno with a free-listing task administered to their parents [10]. In this study, the evidence on actual food consumption comes from an adult, not from the child themselves, a common methodological limitation in the literature on child dietary assessment. Instruments such as the 24-h recall administered directly to the child could help overcome this limitation, provided they are combined with an independent measure of the child’s dietary perception.

Free listing is a projective technique from cognitive anthropology that has been introduced into the food science literature as a tool for obtaining an initial understanding of a food category [11]; it allows researchers to capture the mental structure and cultural salience of foods through the frequency and order in which they are mentioned [12,13]. Specifically, among children, this technique has been successfully applied to identify the ultra-processed snacks with the highest cultural salience among Brazilian schoolchildren, highlighting the role of packaging and marketing in shaping that salience [14]. To the best of our knowledge, no study in the southern Andes region of South America has combined this technique with a 24-h recall reported on the same day by the child, which would allow for a direct test of whether children’s ability to recognize healthy foods is associated with their actual recent intake, without relying on an adult’s report.

In this context, the primary outcome of this study was to determine whether children’s perceptions of food are associated with their actual recent intake. As secondary objectives, providing the necessary basis for testing this primary outcome, the study also aimed to (i) characterize the cultural salience of healthy and unhealthy foods as perceived by schoolchildren at an educational institution in Moquegua, southern Peru, using free-listing and Smith’s salience index, and (ii) describe the children’s self-reported intake of breakfast and lunch using a 24-h recall. These findings aim to provide methodological and substantive evidence on the relationship between perception and eating behavior in childhood, a link with direct implications for the design of nutrition education strategies in similar contexts.

## 2. Materials and Methods

The study was approved by the Ethics Committee of the Technological University of Peru (3347-2026-CEI-UTP) and included informed consent from the parents or guardians and informed assent from the participating children.

### 2.1. Children

The study population consisted of 129 school-aged boys and girls (77 boys and 52 girls, aged 8 to 12 years, with 86.8% of the sample between 9 and 11 years old), students at the “Manuel C. de la Torre” Educational Institution in Moquegua, selected through non-probability convenience sampling. The distribution of gender by age is shown in Table 1.

The sample size (*n* = 129) far exceeded the conventional minimum of 30 participants recommended for obtaining reliable results in free-listing tasks [15] and was comparable to that of similar studies that have applied this technique to children on topics related to eating [16].

### 2.2. Procedure

Prior to data collection, a pilot test was conducted with 30 children of the same age range (8–12 years) at a different school to verify comprehension of the instructions and questions for both the free-listing task and the 24-h recall. The validity of free listing as a technique for eliciting food-related categories in children is further supported by established methodological literature [15,16]. During the pilot, the free-listing task was initially open-ended; however, some children listed more than five foods per domain, introducing excessive variability. Based on this observation, the free-listing task was adjusted to a maximum of five foods per domain for the main data collection.

Data collection was conducted in groups by classroom. In each classroom, two trained researchers provided verbal instructions to the entire group simultaneously, and each child individually and independently completed a printed record sheet. Data collection took place on a regular school weekday, and the 24-h recall referred to the previous day, which was also a regular school weekday, not a holiday or weekend. To control for a potential order effect resulting from the sequence of administration, the participating sections were assigned to one of two sequences: in 3rd and 4th grade classrooms (*n* = 60), the children first completed the 24-h recall (breakfast and lunch from the previous day) and then the free-listing task (five healthy foods and, separately, five unhealthy foods); in 5th grade classrooms (*n* = 69), the order was reversed, beginning with the free-listing task and ending with the 24-h recall. After completing the free-listing task and the 24-h recall, children answered a brief, structured, self-administered questionnaire to characterize their food environment, including the number of people in the household, with whom the child usually has breakfast and lunch, the usual place of food consumption (dining area, kitchen, or restaurant), screen use (TV, phone, or tablet) while eating, and who prepares food in the household.

#### 2.2.1. Free Listing

Each child was asked to write down five foods they considered healthy and, separately, five foods they considered unhealthy, with no time limit and no suggestions from the researcher. Responses were recorded in the order in which they were written, allowing for the calculation of Smith’s cultural salience index [12,13], which was calculated manually using Microsoft Excel spreadsheets.

#### 2.2.2. The 24-Hour Recall

The child was asked to recall and write down what he or she had eaten for breakfast and lunch on the day before the interview. The instrument consisted of a single-pass 24-h recall, with two fixed and identical questions for all participants (breakfast and lunch from the previous day), without any additional probing by the researcher regarding ingredients, preparation, or quantities. Data collection took place on a regular school weekday, and the 24-h recall referred to the previous day, which was also a regular school weekday, not a holiday or weekend.

#### 2.2.3. Brief Food Environment Questionnaire

A brief, structured, self-administered questionnaire was applied to characterize the child’s food environment, including the number of people in the household, with whom the child usually has breakfast and lunch, the usual place of food consumption (dining area, kitchen, or restaurant), screen use (TV, phone, or tablet) while eating, and who prepares food in the household.

### 2.3. Data Analysis

Descriptive statistics (frequencies and percentages) were calculated for the 24-h recall data and for the sociodemographic characteristics of the sample.

For the free-listing data, the cultural salience of each food item was calculated using the Smith index [12,13], which weights the frequency of mention of an item according to the position in which it was recalled by each respondent. The index is defined by Equation (1):

(1) $$S_{i}=\frac{1}{N}\sum_{k=1}^{N}\frac{L_{k}-r_{ik}+1}{L_{k}}$$

where $S_{i}\;$ = salience index of food item *i*; N = total number of respondents (*n* = 129); $L_{k}\;$= number of items mentioned by respondent *k* (maximum 5, according to the open-ended list design); and $r_{ik}$ = position (rank) at which respondent *k* mentioned food item *i* (1 = first food item mentioned). The sum (∑) is calculated across all informants who mentioned food item *i*; if an informant did not mention it, their contribution to the sum is 0. This index ranges from 0 to 1: values close to 1 indicate that a food was mentioned by almost all participants and appeared near the top of the list (greater cultural salience); values close to 0 indicate infrequent mentions or mentions toward the end of the list.

In addition, a cross-analysis was conducted between the results of the free-listing task and the 24-h recall, comparing the average number of healthy foods listed among those who reported actual consumption of healthy/unhealthy foods and those who did not, in order to explore the association between perception and dietary behavior. This analysis was replicated by sex and by order of administration (to verify the absence of an order effect).

To classify responses from the 24-h recall as “healthy” or “unhealthy,” a combined criterion was used based on the degree of food processing according to the NOVA classification [17] and the recommendations of the Dietary Guidelines for the Peruvian Population [18]. Home-cooked meals made with fresh or minimally processed foods and without frying (soups, stews, Andean grains, fruits) were classified as healthy; meals dominated by ultra-processed foods (soda, packaged snacks, processed meats) or fried foods (fried chicken, French fries, pork rinds) were classified as unhealthy. Each dish reported by the children was reviewed and classified individually by the research team, which had direct knowledge of regional culinary traditions; cases with insufficient information for an accurate classification (3.1% of the responses) were recorded as mixed.

Since individual lunch and breakfast dishes showed high variability among participants (129 virtually unique responses per meal), each dish was grouped into broader descriptive categories based on its preparation method and composition, for example, “stews and casseroles,” “fried foods,” “soups or broths,” “grains and pseudograins,” and “fast food or ultra-processed foods,” in order to facilitate a more interpretable frequency analysis. This second categorization is independent of the NOVA classification (healthy, unhealthy, mixed) described in the previous paragraph: while the NOVA classification evaluates the nutritional value of the dish, this categorization groups dishes by type of culinary preparation; for example, a stew and a soup may both be classified as “healthy” in NOVA but belong to different preparation categories. Both classifications were carried out by the research team using the same criteria based on regional culinary knowledge.

Comparisons between independent groups (gender, order of administration) for the number of items listed were performed using the Mann–Whitney U test, since the data did not meet the assumption of normality. Comparisons of categorical variables (healthy/unhealthy consumption, yes/no) were performed using the chi-square test of independence. As an additional robustness check, multivariable logistic regression models were fitted to predict actual healthy food consumption from the free-listing salience score, including a model adjusting for grade level to account for the classroom-based data collection structure. As an additional validity check, data completeness for the free-listing task and the 24-h recall was compared between younger (8–9 years) and older (10–12 years) children using the Mann–Whitney U test and proportion comparisons.

As an exploratory analysis, chi-square tests were also used to examine potential associations between food environment variables (Section 3.8) and actual unhealthy food consumption. A Multiple Correspondence Analysis (MCA) was additionally conducted as a complementary multivariate exploration of the associations between food environment variables and actual dietary intake, using the FactoMineR [19] and factoextra [20] R packages. The significance level was set at *p* < 0.05. All statistical analyses and graphical visualizations were performed using R software version 4.5.3 [21] and the ggplot2 package [22] for generating figures.

## 3. Results

### 3.1. Perception of Healthy Foods (Free Listing)

The foods that participants spontaneously associated with a healthy diet were recorded. Figure 1 shows the 15 foods with the highest cultural salience (Smith index); the percentage next to each bar indicates the percentage of children who mentioned it. The apple was the food with the highest cultural salience in the “healthy eating” domain (S = 0.334, mentioned by 47.3% of participants), followed by carrots and bananas. There is a clear predominance of fruits and vegetables that are easily accessible and consumed daily in the region, while the mention of “water” as part of a healthy diet was relatively low (7.8%).

The bars in Figure 1 are ranked according to Smith’s salience index (S), which weights both the frequency of mention and the position in which each food was mentioned, while the percentage next to each bar reflects only the frequency of mention; therefore, the two rankings do not always coincide. For example, cucumber was mentioned more frequently than mango or fruit salad (16.3% vs. 12.4% and 11.6%, respectively), but with lower salience (S = 0.075 vs. S = 0.084 and S = 0.086), indicating that the latter tended to be mentioned earlier (with higher cognitive priority) in the children’s lists.

Although the open-ended list was completely open-ended, with no restrictions or suggestions from the researcher, other food groups were also mentioned by the children, albeit with considerably less frequency than fruits and vegetables: hard-boiled eggs (*n* = 4), cheese (*n* = 1), chicken (*n* = 3), meat (*n* = 2), fish (*n* = 3), and lentils (*n* = 3), out of a total of 106 different foods mentioned across the entire sample. This pattern suggests that the cognitive representation of “healthy food” in this population is centered predominantly on fruits and vegetables, while protein sources and dairy products occupy a comparatively marginal position.

### 3.2. Perception of Unhealthy Foods (Free Listing)

Figure 2 shows the 15 foods with the highest salience in the “unhealthy” domain. Soda was the item with the highest salience in the “unhealthy eating” domain (S = 0.311, 46.2% of mentions), followed by French fries and hamburgers. It is noteworthy that “grilled chicken” and “fried chicken” (traditional dishes frequently consumed by families in Peru) appear with considerable salience within the “unhealthy” category (S = 0.120 and 0.105, respectively), suggesting that children associate the method of preparation (fried or roasted) rather than the base food itself with the “unhealthy” category.

### 3.3. Twenty-Four-Hour Recall Breakfast

When all variants were grouped together (alone, as a juice, with bread, or combined with other ingredients), quinoa was the most frequently reported individual food item for breakfast, present in 19 of 128 responses (14.8%). It was followed, in descending order of frequency, by Quaker (8), rice with a fried egg (5), broth (4), Quaker with bread (4), cereal with yogurt (4), and, less frequently, grilled chicken, milk, bread with sausage, papaya juice, arepa, and coffee (2 each).

The reported breakfasts were grouped into broader categories based on their composition (Table 2).

Cereals (oats) and traditional Andean beverages such as quinoa (Chenopodium quinoa), maca (Lepidium meyenii Walpers), and api (purple corn flour) are present as a breakfast staple; together, they account for 37.5% of the sample. The persistence of these local eating patterns is striking given the growing availability of processed food options. The category “beverage with traditional Torata bread” (coffee, tea, or mate, generally accompanied by traditional local bread without a substantial filling) was the third most frequent (14.8%), followed very closely by fried foods (14.1%). The ultra-processed and “bread with cold cuts” categories, combined, accounted for an additional 11.6% of the reported breakfasts. Traditional Torata bread is clearly distinct from industrial sliced bread, reflecting the coexistence of local artisanal baking and industrial bakery products in these schoolchildren’s diets.

### 3.4. Twenty-Four-Hour Recall Lunch

The dishes most frequently reported as the previous day’s lunch were fried chicken (6), green rice (5), stir-fry (5), stew (4), broth (4), green noodles (4), chaufa rice (4), and, less frequently, lentils, rice with chicken, ceviche, pumpkin locro, stew, and rice with egg (3 each).

Since individual lunches vary greatly from day to day and from person to person (129 responses were unique with little repetition), the dishes were grouped into broader categories based on their method of preparation (Table 3), which allows for a more robust analysis than simply counting the frequency of individual dishes.

Nearly a quarter of the reported lunches (23.4%) consisted of fried foods, while one-third (32%) consisted of stews, braised dishes, or roasts that were not fried (traditional homemade dishes from the region). Pasta accounted for an additional 14.1%, and categories strictly associated with fast food or ultra-processed foods (hamburgers, pizza) accounted for only 5.5% of lunches. This indicates that traditional home-cooked lunches remain predominant over fast food, although fried foods are still quite common.

It is important to note that this preparation-type categorization is independent of the NOVA-based healthy/unhealthy/mixed classification used later (Section 3.6 and Section 3.7): While the present categorization describes what type of dish the child consumed based on its cooking method, the NOVA classification evaluates the nutritional quality of that same response, used specifically to test the study’s central hypothesis. Both classifications are complementary rather than redundant: for example, a “stew/braised dish” was almost always classified as healthy under NOVA, whereas a “fried” preparation was almost always classified as unhealthy.

### 3.5. Differences by Gender

The average number of foods mentioned in the open-ended list was compared between boys and girls for both the “healthy foods” and “unhealthy foods” categories using the Mann–Whitney U test (Table 4).

No statistically significant differences were observed between girls and boys in the number of foods recalled, either in the healthy domain (*p* = 0.992) or in the unhealthy domain (*p* = 0.343).

#### 3.5.1. Types of Foods Mentioned in the Healthy and Unhealthy Lists, by Sex

The foods from the free-response list were classified by type (fruits, vegetables, sweets/snacks, and fast food, among others) to explore whether there were gender differences in the specific content of each category, beyond the total number of items mentioned (Table 5).

In the “healthy” category, there are no significant differences: both girls and boys mention fruits and vegetables in very similar proportions. In the “unhealthy” category, boys mention fast food slightly more often than girls (33.9% versus 30.3% of their mentions), although the difference is small, and both groups agree that sweets/snacks are the predominant category.

#### 3.5.2. Reported Actual Consumption (24-Hour Recall)

Reported actual consumption was also compared between girls and boys using the NOVA classification and the chi-square test of independence (Table 6).

Figure 3 visually summarizes this contrast; the numerical means differ slightly between the sexes, but neither comparison reached statistical significance (*p* = 0.912 and *p* = 0.603, respectively), likely due to the sample size per group.

### 3.6. The Intersection of Perception and Actual Practice

We explored whether children who listed more healthy foods in the open-ended list also reported healthier actual consumption at breakfast or lunch the previous day, using the final manual classification based on the NOVA classification and the Peruvian Dietary Guidelines. The Man–Whitney U test (*n* = 128, participants with complete 24-h recall data). 85.2% of the children reported at least one food classified as healthy in their breakfast or lunch from the previous day. 38.3% reported at least one food classified as unhealthy. The average number of healthy foods mentioned in the free-listing task was identical among those who actually ate healthily (average 4.93 items, *n* = 109) and those who did not (average 4.84 items, *n* = 19), with no statistically significant difference (U = 1072.0, *p* = 0.633).

As an additional robustness check, a multivariable logistic regression was fitted to predict actual healthy food consumption from the free-listing score, adjusting for age and sex (*n* = 128); the association remained non-significant after adjustment (OR = 2.03, 95% CI: 0.57–7.20, *p* = 0.272). Because data collection occurred by classroom, we tested whether classroom-level clustering influenced this result. A model clustering by the five individual classrooms was unstable due to the very small number of clusters, and a fixed-effects model using individual classrooms as a covariate failed to converge due to quasi-complete separation in the smallest classroom (*n* = 9, 100% reporting healthy intake). An alternative model adjusting for grade level (3rd/4th/5th grade) and sex converged successfully and confirmed the null association (OR = 1.88, 95% CI: 0.54–6.52, *p* = 0.320). However, the wide confidence interval reflects considerable uncertainty in the effect estimate, given the available sample size; this result should therefore be interpreted as consistent with the absence of association observed in the main analyses, rather than as conclusive evidence that the finding is robust to classroom-level grouping.

### 3.7. Verification of the Order Effect

Participants were divided into two administration sequences based on classroom. Sequence 1 consisted of 3rd and 4th graders, who completed the 24-h recall first and then the free-listing (*n* = 60), while Sequence 2 consisted of 5th-grade students, who completed the free-listing first and then the recall (*n* = 69 recruited, *n* = 68 with complete 24-h recall data). A chi-square test was used to determine whether the order of administration influenced the results (Table 7). 

The percentage of children who reported at least one unhealthy food item was virtually identical between the sequence that began with the 24-h recall (38.3%) and the one that began with the free-listing method (38.2%, χ^2^ = 0.000, *p* = 1.000). This result provides no evidence that the order in which the instruments were administered influenced the observed gap between perception and actual consumption.

### 3.8. Food Environment

To characterize children’s food environment, a brief complementary questionnaire was applied (*n* = 129). The average household comprised 4.1 people (median 4; range 2–6). Most children reported having breakfast (89.1%) and lunch (84.5%) accompanied, rather than alone. Meals were predominantly consumed in the household dining area (85.3%), followed by restaurants (14.0%) and the kitchen (0.8%). The large majority of children (90.7%) reported using screens (TV, phone, or tablet) while eating. Regarding food preparation, the mother was the main person responsible (76.7%), followed by a sister (7.8%), a domestic employee (6.2%), the grandmother (4.7%), and the father (4.7%).

As an exploratory analysis, children who had lunch alone reported a notably higher rate of unhealthy food consumption than those eating with someone (60.0% vs. 34.3%), a difference approaching the conventional significance threshold (χ^2^ = 3.706, *p* = 0.054, *n* = 20 vs. *n* = 108). A similar, weaker pattern was observed for breakfast (alone: 57.1% vs. accompanied: 36.0%; χ^2^ = 1.555, *p* = 0.212, *n* = 14 vs. *n* = 114). No relevant differences were found according to screen use while eating (with screen: 37.1% vs. without: 50.0%; χ^2^ = 0.320, *p* = 0.572) or place of consumption (dining area: 38.5% vs. elsewhere: 36.8%; χ^2^ = 0.000, *p* = 1.000). These exploratory results should be interpreted with caution given the small size of some subgroups but consistently suggest that eating with others, particularly at lunch, may be associated with lower unhealthy food consumption, a hypothesis that future adequately powered studies should confirm.

As a complementary multivariate visualization, a Multiple Correspondence Analysis (MCA) was conducted that included sex, actual healthy/unhealthy consumption, and food environment variables (Figure 4). The first two dimensions explained 44.3% of the total variance (25.4% and 18.9%, respectively). The resulting map showed a clear pattern along the first dimension: categories reflecting accompanied eating (breakfast and lunch) and healthy consumption clustered together, opposite to categories reflecting eating alone and unhealthy consumption, visually reinforcing the exploratory associations reported above.

### 3.9. Instrument Validity by Age Group

Given that the sample included children aged 8 to 12 years, data completeness and quality were compared between the younger group (8–9 years, *n* = 39) and the older group (10–12 years, *n* = 90). The mean number of items listed in the free-listing task (maximum 5) did not differ significantly between groups (8–9 years: mean = 4.95; 10–12 years: mean = 4.90; U = 1823.0, *p* = 0.492). Completeness of the 24-h recall was high and comparable across groups (100.0% vs. 97.8%). These results support the validity of analyzing children aged 8 to 12 together in this study.

## 4. Discussion

Apples, carrots, and bananas ranked highest in cultural salience within the “healthy” domain; all are fruits and vegetables that are inexpensive and widely available in local markets in the southern Andes region of Peru. This is consistent with the approach of Albardan and Platat [4] and Nansel et al. [23], in which children’s food preferences and categorizations are progressively constructed through repeated exposure to foods in their immediate environment, rather than through abstract nutritional reasoning. The low relative salience of water (S = 0.057) indicates that children associate “healthy eating” primarily with solid, visible foods, a pattern also reported by Scaglioni et al. [3] when analyzing the factors that shape children’s eating behavior. The representation of “healthy food” in this sample was dominated almost exclusively by fruits and vegetables, with only a marginal presence of proteins and dairy products. This may reflect the fact that school nutrition education programs tend to focus specifically on promoting fruits and vegetables, rather than on a broader concept of a balanced diet [24]. This suggests that educational strategies in the region could benefit from explicitly incorporating the role of proteins and dairy products.

A particularly relevant finding was that children associated dishes such as grilled chicken and fried chicken (S = 0.120 and S = 0.105, respectively), which are frequently consumed by families and culturally valued in Peru, with the “unhealthy” category, indicating that children’s classification of “unhealthy” is based more on the method of preparation (fried, roasted in fat) than the staple food itself. This observation reinforces the approach of Albardan and Platat [4] regarding the role of sensory associations (texture, fat) in children’s food categorization and is consistent with [3], who note that these judgments appear to occur largely independently of formal nutritional knowledge.

The continued presence of Andean grains and traditional beverages (quinoa, maca, and api) as the basis for nearly 38% of reported breakfasts, along with the predominance of stews and homemade, non-fried dishes for lunch (32.0%, compared to only 5.5% fast food and ultra-processed foods), suggests that the daily diet of these schoolchildren largely preserves regional eating patterns, in contrast to the trend toward dietary Westernization documented in other middle-income urban contexts [6,9]. However, the presence of fried foods (23–24% at both meals) indicates that this dietary transition is also occurring, albeit to a more moderate extent.

No significant differences were observed between boys and girls in either the quantity or the type of foods mentioned in the free-response list (Table 4 and Table 5), indicating that the cognitive structure of food perception is equivalent between the sexes in this sample. However, a non-significant numerical difference was observed in the actual consumption of unhealthy foods (boys: 40.8%, girls: 34.6%; *p* = 0.603). If confirmed in future studies with greater statistical power, this trend would align with the findings reported by Shaw et al. [25], who noted that factors external to the individual (access to and exposure to fast-food establishments and varying levels of autonomy in making purchases) may influence actual eating behavior, beyond individual differences in knowledge or perception.

No association was found between children’s perceptions of food and their recent actual intake (U = 1072.0, *p* = 0.633), a result that remained consistent when three different criteria for classifying actual consumption were applied. This finding is consistent with the literature on psychosocial correlates of children’s eating behavior, in which nutritional knowledge and perceived food availability did not show a consistent relationship with actual consumption [26]. Similarly, a population-based study of Polish adolescents found that nutritional knowledge only partially explains the quality of actual diet, once sociodemographic and lifestyle variables are taken into account [27], and a recent systematic review on eating behaviors in children and adolescents confirmed that eating behavior patterns predict actual intake more consistently than isolated measures of knowledge [28]. Our results extend this evidence to a Peruvian Andean population that has not previously been studied using this methodological approach.

The fact that both administration sequences yielded virtually identical results (χ^2^ = 0.000, *p* = 1.000) provides no evidence that the order of the instruments influenced the results. This finding is consistent with a gap between perception and eating behavior in this sample, although it should not be interpreted as definitive evidence of a broader or causal pattern beyond the scope of this study’s design.

On the food environment, children who ate lunch alone reported a notably higher rate of unhealthy food consumption than those eating with someone (60.0% vs. 34.3%), a difference that approached the conventional significance threshold (*p* = 0.054). Although exploratory and limited by subgroup size, this pattern is consistent with a substantial body of evidence linking shared family meals to healthier dietary patterns in children and adolescents [29]. This pattern was further supported by a complementary multivariate visualization (MCA, Figure 4), which showed that categories reflecting accompanied eating and healthy consumption clustered together, opposite to categories reflecting eating alone and unhealthy consumption. This finding complements our central result: while food perception alone was not associated with actual intake, the immediate social context of eating showed a numerically larger, though still not statistically significant, association with healthy consumption.

These results are part of a broader line of research by our group on children’s dietary perceptions in the southern Peruvian Andes. Unlike our previous study, which assessed children’s perceptions using a structured sorting task and obtained information on healthy/unhealthy foods from parents [10], the present study is the first in this line of research to obtain both perceptions and actual intake directly from the children themselves, without adult mediation. This methodological feature allows for a more direct test of the gap between knowledge and practice than designs focused exclusively on parental reporting.

This study has several limitations. First, the cross-sectional design precludes establishing causal relationships between perception and consumption. Second, the sample was drawn from a single school in Moquegua, selected by convenience, limiting generalizability to other populations in southern Andean Peru. Third, the 24-h recall was self-reported by children without adult verification, which may introduce recall errors, particularly among younger children (8–9 years). Additionally, the recall was administered only once per participant and therefore reflects a single day’s intake rather than children’s habitual dietary patterns, which would require multiple non-consecutive recall days, including at least one weekend day; findings regarding “actual intake” in this study should be interpreted strictly as recent reported intake on the day preceding the interview. Furthermore, the recall only assessed breakfast and lunch and did not capture dinner or between-meal snacks; therefore, “actual intake” in this study should be understood as partial daily intake, not a complete account of the child’s full dietary day. Fourth, the free-listing task was capped at five foods per domain, which may have truncated some children’s full food repertoire. Fifth, the food environment questionnaire (Section 3.8) used simple, non-validated items, and its exploratory associations with actual intake were limited by small subgroup sizes (e.g., *n* = 20 eating lunch alone); these findings, including the marginal association with unhealthy consumption (*p* = 0.054), should be considered preliminary. Data collection involved two trained researchers following a standardized protocol; however, interviewer identity was not recorded per participant, precluding a formal statistical comparison of results by interviewer. Additionally, this study did not directly assess children’s specific sources of nutrition-related information (school, internet, television, family); only screen use during meals was measured, which captures media exposure only partially.

These findings suggest that nutrition education strategies in this population should extend beyond knowledge transmission alone, incorporating family-level interventions, school food environment regulation (e.g., school kiosk food offerings), and effective enforcement of Peru’s existing food policy framework (Ley 30021) at the point of purchase. This is further supported by our own exploratory finding that children eating lunch with others reported lower unhealthy food consumption (Section 3.8), reinforcing that family-level factors, not just individual knowledge, should be a central target of intervention.

## 5. Conclusions

Schoolchildren in Moquegua, southern Peru, clearly identify which foods are culturally perceived as healthy (led by apples, carrots, and bananas) and which are perceived as unhealthy (led by soda, French fries, and hamburgers), but this ability to distinguish between them was not associated with their actual consumption reported the previous day (*p* = 0.633). This finding constitutes the first direct evaluation, in this line of regional research, of the relationship between children’s food knowledge and daily eating practices when both are measured in the same child; in this sample, no statistically significant association was detected between them.

These results have direct practical implications: nutrition education programs targeting schoolchildren in the Peruvian Southern Andes should not assume, based solely on these findings, that teaching children to recognize healthy foods is necessarily sufficient to change their actual diet. It is necessary to complement the transmission of knowledge with strategies that address the family environment, food availability at home and school, and children’s actual opportunities for food choice. Future studies in this line of research could expand the sample to include other educational institutions in the southern Peruvian Andes, incorporate a longitudinal component to examine whether the absence of association observed here between perception and practice persists or changes with age, and explore the specific role of the family and the school environment as mediators between children’s nutritional knowledge and actual food consumption.

## Figures and Tables

**Figure 1:**
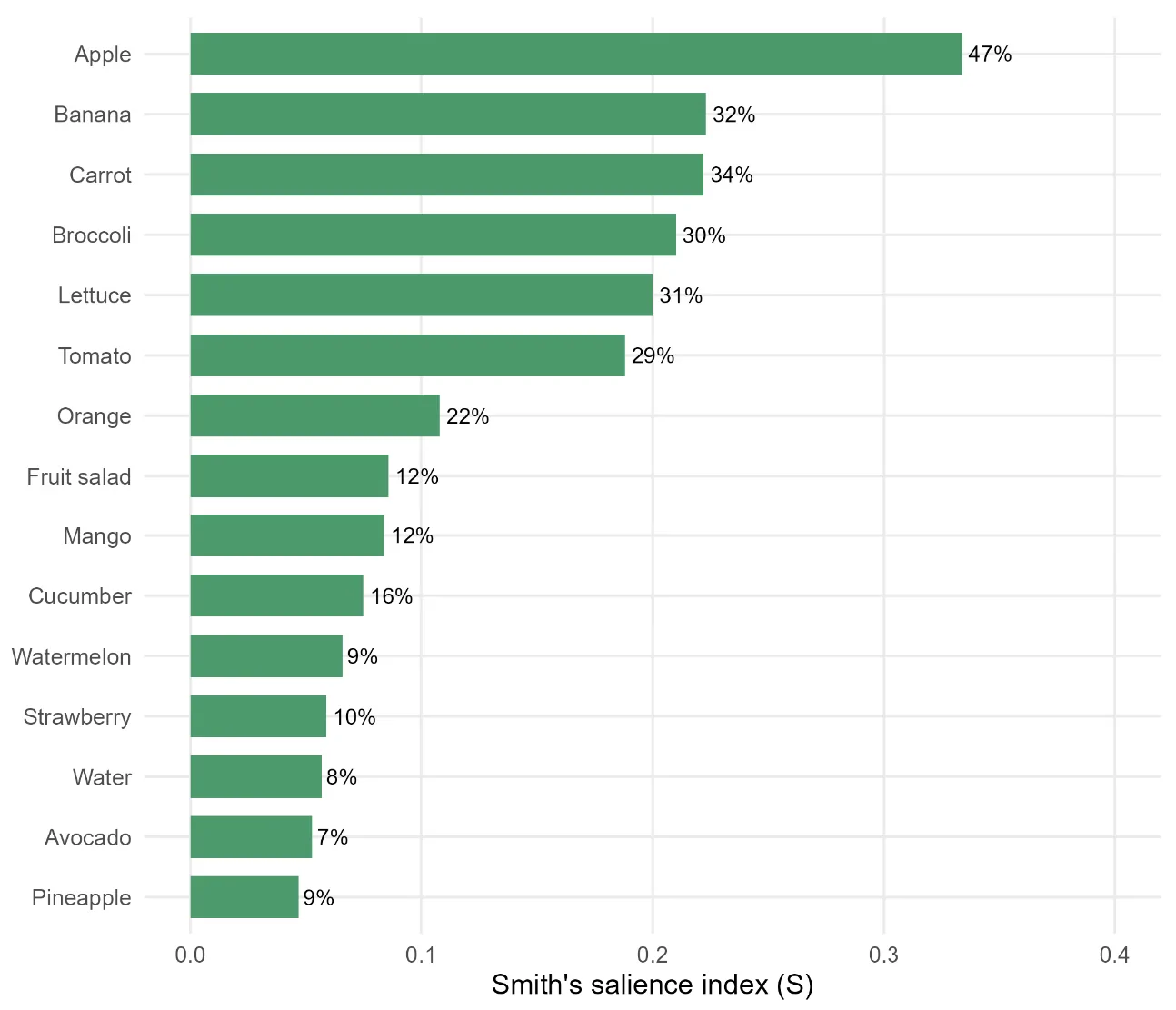
Salience of healthy foods.

**Figure 2:**
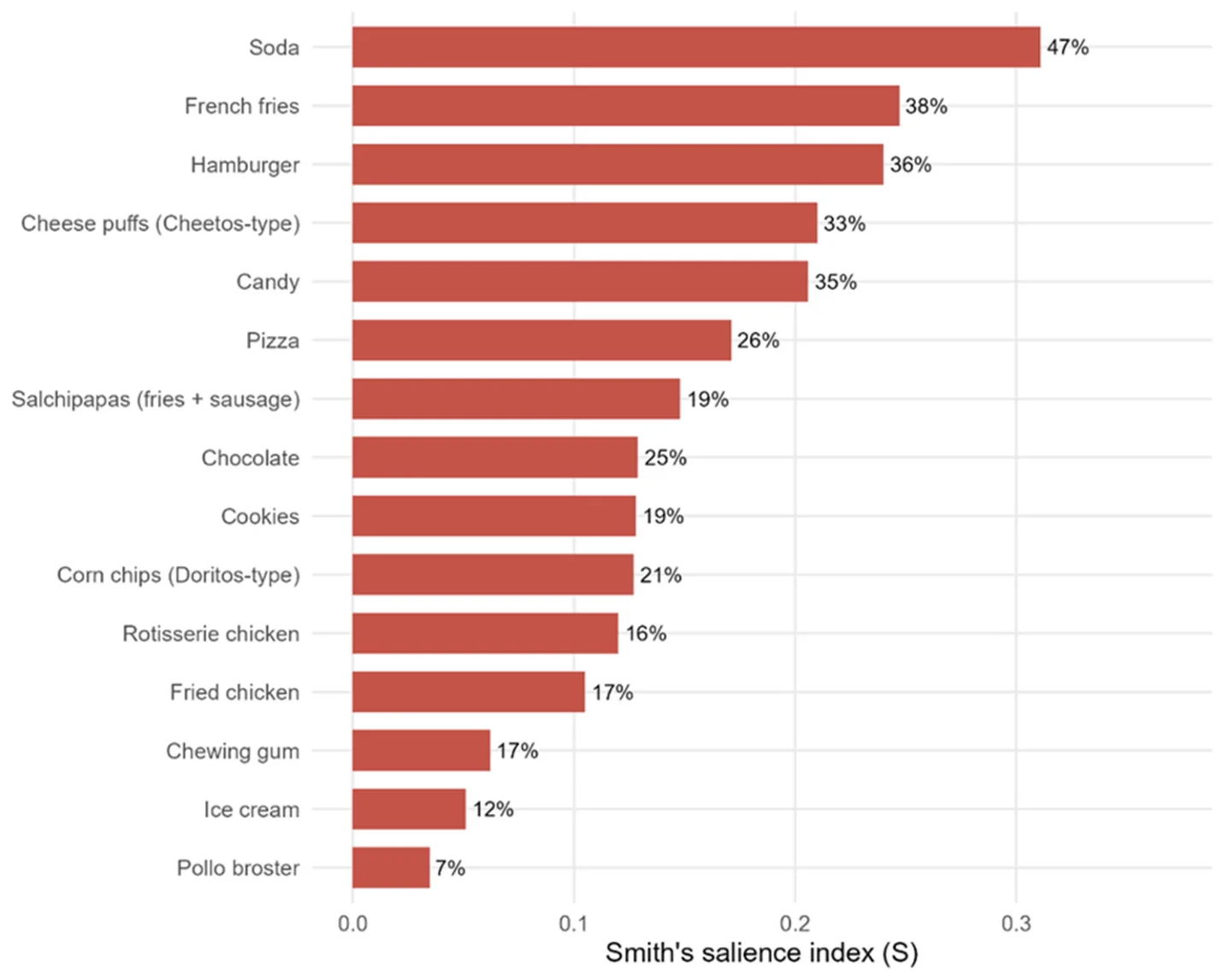
Salience of unhealthy foods.

**Figure 3:**
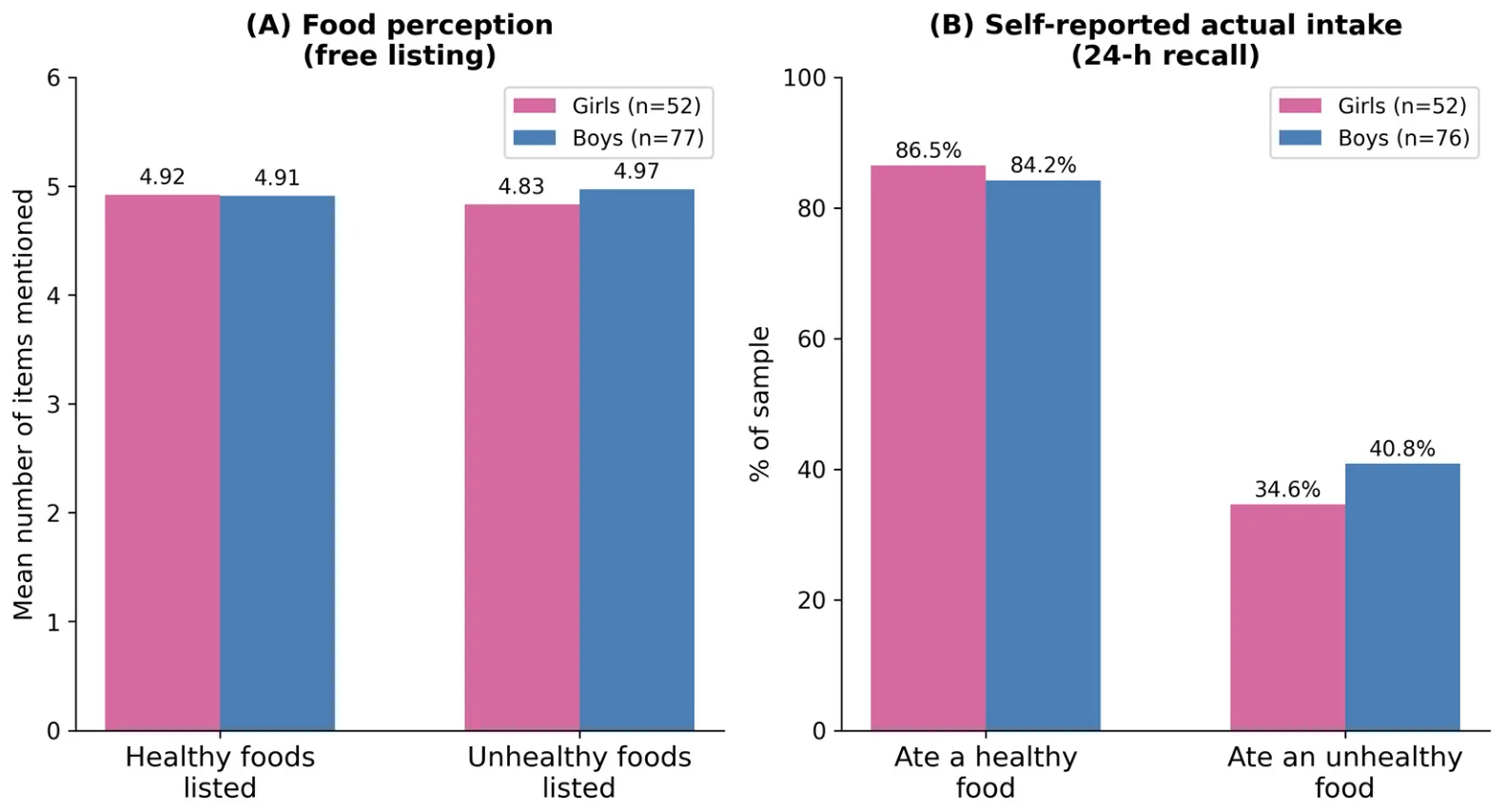
Similar perception between sexes vs. different actual intake: (**A**) Food perception assessed via free listing (mean number of items mentioned). (**B**) Self-reported actual dietary intake via 24-h recall.

**Figure 4:**
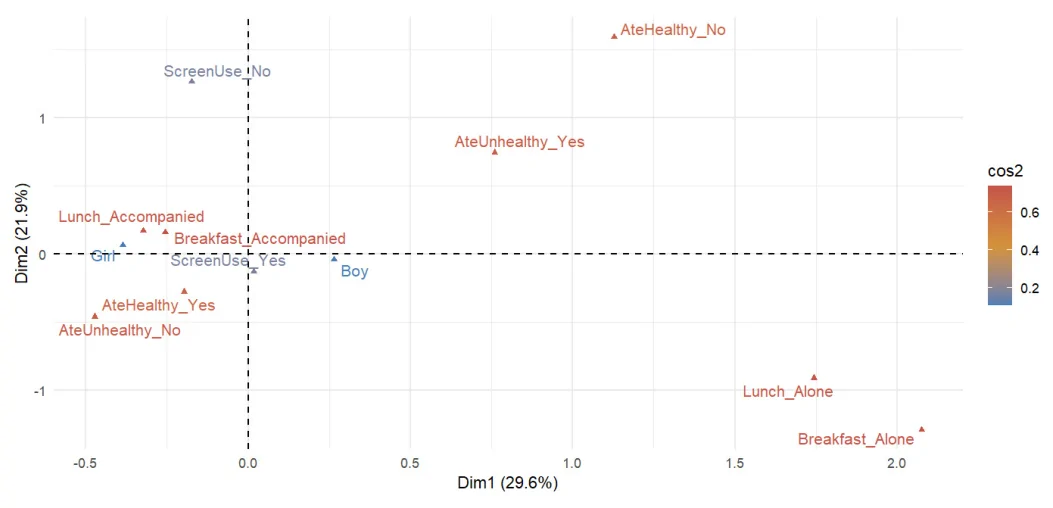
Multiple Correspondence Analysis (MCA) of sex, actual dietary intake, and food environment variables (*n* = 128). Colors represent the cos^2^ value (squared cosine), indicating the quality of representation of each category on the two dimensions shown; categories in orange/red are better represented by these two dimensions than those in blue.

**Table 1:** Distribution of gender by age (*n* = 129).

Age	Girls	Boys	Total
**8 years**	10	6	16
**9 years**	6	17	23
**10 years**	21	30	51
**11 years**	15	23	38
**12 years**	0	1	1
**Total**	52	77	129

**Table 2:** Composition categories of reported breakfasts (*n* = 128).

Category	Frequency	%
Cereals (Quaker, oats, cereal)	27	21.1
Traditional Andean beverages (api, maca, quinoa)	21	16.4
Beverages and traditional Torata bread (coffee, tea, mate, either on their own or with traditional bread without a substantial filling)	19	14.8
Fried foods (fried eggs, fried fish, grilled/broasted chicken)	18	14.1
Ultra-processed/sweet (chocolate, alfajor, panettone, packaged snacks)	8	6.2
Fruit and dairy (milk, fruit juice, fruit salad)	7	5.5
Bread with cold cuts (ham, chorizo, sausage)	7	5.5
Bread with protein (egg, tuna, meat, not fried)	6	4.7
Soups, broths	6	4.7
Juice + bread with healthy fillings (avocado, cheese)	6	4.7
Bread and refined grains (white bread, sliced bread)	2	1.6
I don’t remember	1	0.8

Note: Traditional local bread (bread Torata) refers to the traditional artisanal bread from the Torata district (Mariscal Nieto province, Moquegua), which is consumed daily in the region. The “Traditional Andean beverages” category includes 15 mentions of quinoa, plus api (1), maca (4), and cañihua (1).

**Table 3:** Preparation categories of reported lunches (*n* = 129).

Category	Frequency	%
Stews, braised dishes/roasts (non-fried protein)	41	32.0
Fried foods (fried chicken/fish/cheese, pork rinds, salchipapa)	30	23.4
Pasta (tallarín, spaghetti, noodles)	18	14.1
Others	12	9.4
Soups, broths	10	7.8
Legumes, grains (lentils, beans, barley)	7	5.5
Fast food/ultra-processed foods (hamburgers, pizza)	7	5.5
Ceviche (raw fish)	3	2.3

**Table 4:** Average number of items listed by gender (free listing).

Average	Girls (*n* = 52)	Boys (*n* = 77)	Statistic	*p*
Number of healthy foods listed	4.92	4.91	U = 2003.0	0.992
Number of unhealthy foods listed	4.83	4.97	U = 1935.5	0.343

**Table 5:** Categories of healthy and unhealthy foods listed by gender.

Listed	Category	Girls (% of Mentions)	Boys (% of Mentions)
**healthy foods**	Fruits	33.6%	36.5%
	Vegetables	30.5%	31.0%
	Others (water, grains, legumes, etc.)	34.4%	31.0%
**unhealthy foods**	Treats, snacks (soda, candy, Cheetos, cookies)	44.6%	43.1%
	Fast food (hamburgers, pizza, fried chicken, hot dogs, and fries)	30.3%	33.9%
	Others	25.1%	23.0%

**Table 6:** Reported actual consumption by sex (24-h recall).

	Girls (*n* = 52)	Boys (*n* = 76)	Statistic (χ^2^)	*p*
**% who reported healthy food in their actual meal**	86.5%	84.2%	0.012	0.912
**% reporting unhealthy food in their actual meal**	34.6%	40.8%	0.271	0.603

**Table 7:** Reported actual consumption by administration sequence.

	Sequence 1 (*n* = 60)	Sequence 2 (*n* = 68)	χ^2^	*p*
Percentage reporting unhealthy food in their actual meal	38.3%	38.2%	0.000	1.000

## Data Availability

The data presented in this study are available upon request from the corresponding author. The data are not publicly available due to ethical and privacy restrictions associated with the participation of minors in the study.
